# Antimicrobial treatment of *Brucella*-infected prosthetic knee with sinus tract: A case report

**DOI:** 10.1097/MD.0000000000046946

**Published:** 2026-01-09

**Authors:** Hongyin Fu, Song Sun, Xiuyuan Wang, Lianggong Zhao, Hu Chen, Rui Bai, Honghao Fu, Libo Zhao

**Affiliations:** a Department of Orthopaedics, The Second Hospital of Lanzhou University, Lanzhou City, China; b Department of Anesthesiology and Surgery, The Second Hospital of Lanzhou University, Lanzhou City, China; c Department of Anorectal Surgery, People’s Hospital of Guangming District, Shenzhen City, China.

**Keywords:** brucellosis, case report, infection, sinus tract, total knee arthroplasty

## Abstract

**Rationale:**

*Brucella* infection of prosthetic joints is exceedingly rare. Current literature indicates that cases complicated by abscess and sinus tract, but without prosthetic loosening, have uniformly been managed with either debridement, antibiotics, and implant retention or revision arthroplasty combined with antimicrobial therapy. These approaches impose a significant economic burden on healthcare systems. Herein, we present a case of *Brucella*-infected prosthetic knee with sinus tract formation and radiographically stable components that was successfully cured with antimicrobial therapy alone.

**Patient concerns:**

A 63-year-old Chinese woman underwent total knee arthroplasty 13 months prior for osteoarthritis. Subsequently, she developed a periprosthetic joint infection and received treatment at a local hospital. Due to atypical clinical symptoms, inconclusive radiographic findings, and negative bacterial cultures, arthrotomy with drainage, exchange of the tibial polyethylene insert, and empirical anti-tuberculosis therapy were performed. However, the infection failed to resolve.

**Diagnoses:**

The patient presented with knee swelling, pain, purulent drainage, and a sinus tract following total knee arthroplasty. A positive standard tube agglutination test, coupled with a history of chronic sheep exposure, confirmed the diagnosis of *Brucella* infection.

**Interventions:**

Since the patient’s prosthesis showed no loosening, a dual regimen of doxycycline and rifampicin was administered for 3 months.

**Outcomes:**

The sinus tract healed completely, standard tube agglutination test serology reverted to negative, and full restoration of knee function was recovered.

**Lessons:**

For patients definitively diagnosed with *Brucella* periprosthetic joint infection and radiographically stable implants, conservative management with antimicrobial therapy alone should be considered as a viable initial treatment option, irrespective of the presence of a sinus tract. Knee prosthesis removal and spacer implantation may be deferred in such cases.

## 1. Introduction

Brucellosis is a zoonotic disease. The global annual incidence of human brucellosis is 2.1 million, with the majority of cases and risk concentrated in Africa and Asia.^[1]^ In China, high-prevalence areas for brucellosis include Inner Mongolia Autonomous Region, Xinjiang Uygur Autonomous Region, Shanxi Province, Heilongjiang Province, Jilin Province, Liaoning Province, Ningxia Hui Autonomous Region, Gansu Province, and Shaanxi Province.^[2]^ Musculoskeletal involvement is the most common complication of brucellosis, occurring in 2% to 77% of *Brucella*-infected individuals. It can manifest during both acute and chronic phases, with clinical presentations including peripheral arthritis, spondylitis, sacroiliitis, and osteomyelitis. Peripheral arthritis primarily affects large joints such as the knee, hip, and ankle joints.^[3]^ A retrospective analysis of 1590 brucellosis patients found that 91.82% developed complications, with arthritis involvement at 62.2%.^[4]^ Shi et al reported a peripheral arthritis involvement rate of 30% in a study of 880 brucellosis patients.^[5]^ The most common clinical symptoms are fever, sweating, fatigue, and joint pain.^[4,5]^ The overall incidence of periprosthetic joint infection (PJI) following total knee arthroplasty (TKA) is 0.83%. Infection is the leading cause of early revision after TKA. The overall random effects model estimated that the incidence of PJI within 1 year and after 1 year post-TKA were 1.11% and 0.41%, respectively.^[6]^ *Brucella* infection of prosthetic joints is rare. Among 278 studies on the microbial distribution of PJI, the overall prevalence was 56.6% for gram-positive bacteria, 28.1% for gram-negative bacteria, 9.3% for fungi, and 0.3% for mycobacteria.^[7]^ Analysis by Mina W. Morcos et al of matching cohorts of 73 patients each underwent revision TKA (mean age 68.8 years, range 48–91 years) or primary TKA (mean age 65.9 years, range 50–86). Significant differences favoring primary TKA were observed: 2-stage revision surgery necessitated a longer mean hospital stay (22.7 days vs 3.84 days, *P* < .001), required more mean outpatient visits (8 vs 3, *P* < .001), led to more readmissions (29 vs 0, *P* < .001), and incurred higher mean total costs ($35,429.97 vs $6809.94, *P* < .001). Therefore, revision treatment for PJI imposes a substantial economic burden on the healthcare system.^[8]^ Consequently, we present a case of a patient with a *Brucella*-infected TKA associated with a sinus tract but with radiographically stable components, successfully treated with antimicrobial therapy alone.

## 2. Case report

A 63-year-old female patient underwent left TKA at a local hospital on May 24, 2023, for severe left knee osteoarthritis. Postoperatively, she developed progressive swelling, pain, and restricted range of motion in the left knee. She reported no fever, sweating, or fatigue. Local skin erythema was absent. Follow-up at the local hospital revealed normal complete blood count, C-reactive protein (CRP), and procalcitonin (PCT), with only mildly elevated erythrocyte sedimentation rate (ESR). Symptomatic management, including anti-inflammatory and analgesic agents, was administered for 5 months without improvement; knee swelling and pain worsened. Repeat ESR remained mildly elevated, while other inflammatory markers were essentially normal. Suspecting a PJI, the local surgeon performed knee arthrotomy with debridement and drainage on October 28, 2023. Intraoperatively, clear yellow fluid was drained. Routine bacterial cultures were negative. Considering possible tuberculosis (TB) infection, antitubercular therapy was initiated. After 2 months of treatment, the surgical wound exhibited persistent drainage, and knee pain and functional impairment worsened.

**Figure 1.**
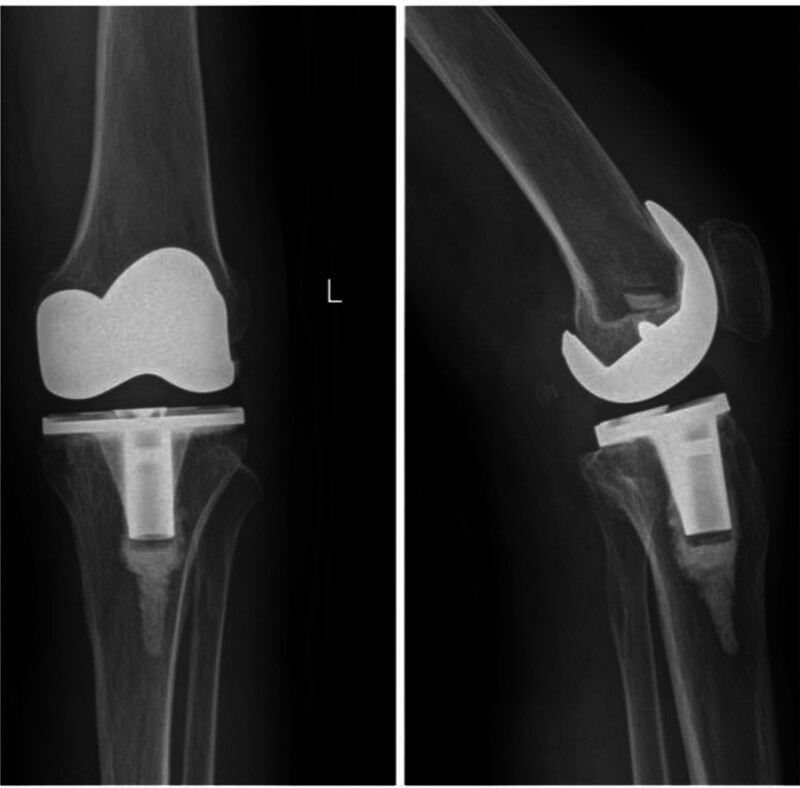
Stable prosthesis positioning without loosening, displacement.

On January 8, 2024, she presented to another local hospital. Blood tests showed: interleukin-6 (IL-6) 4.3 pg/mL, PCT 0.012 ng/mL, white blood cell count (WBC) 5.9 × 10⁹/L, ESR 30 mm/h. Anteroposterior and lateral radiographs of the left knee showed the prosthetic components in situ without evidence of loosening or migration (Fig. 1). Three consecutive cultures of wound discharge and joint fluid were negative. Persisting symptoms led to a continued suspicion of TB infection. A second left knee arthrotomy with debridement, drainage, and exchange of the tibial polyethylene insert was performed. Antitubercular therapy was continued for an additional 7 months. However, left knee swelling, pain, functional limitation persisted, and the inferior aspect of the incision broke down, forming a draining sinus tract.

**Figure 2.**
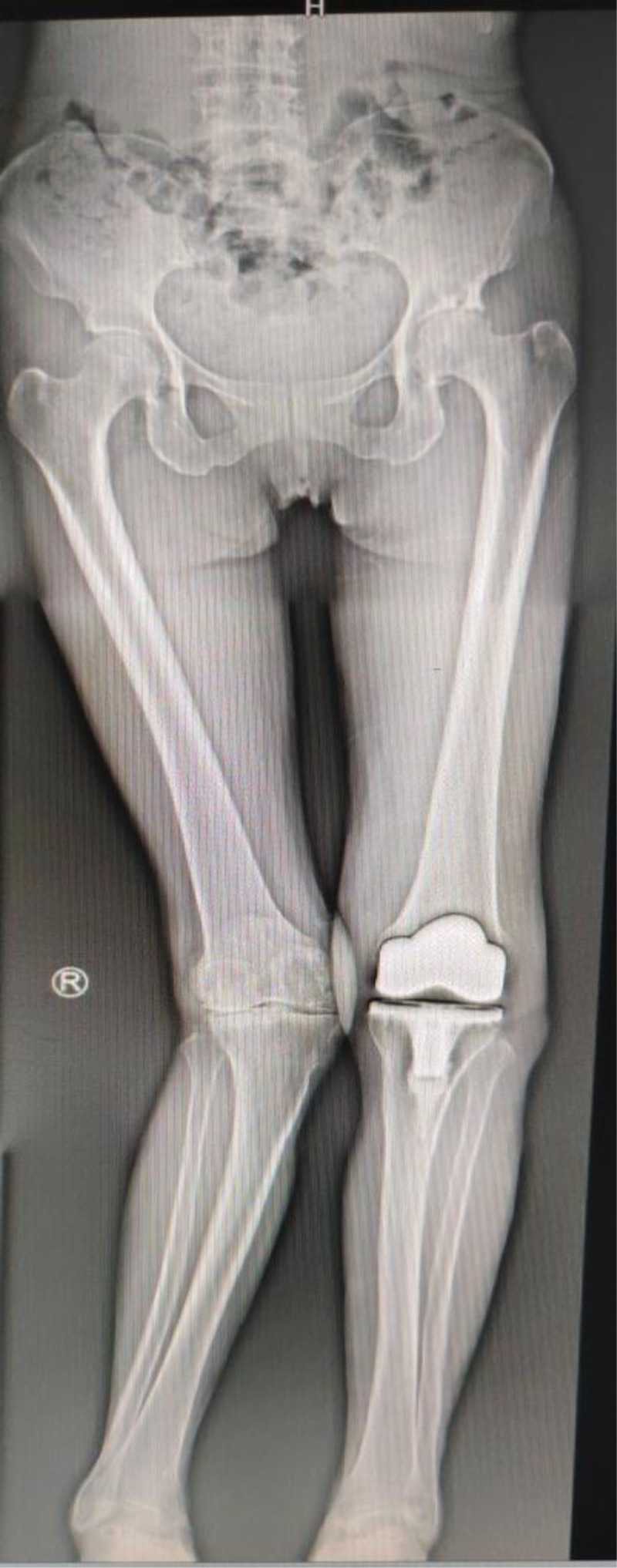
Stable left knee prosthesis, right knee varus deformity with joint space narrowing, and left hip osteoarthritic changes.

**Figure 3.**
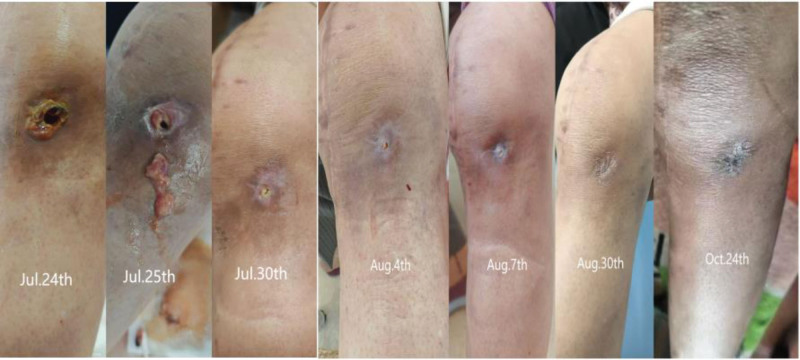
The joint effusion gradually diminishes and resolves completely, while the sinus tract progressively narrows and heals.

**Figure 4.**
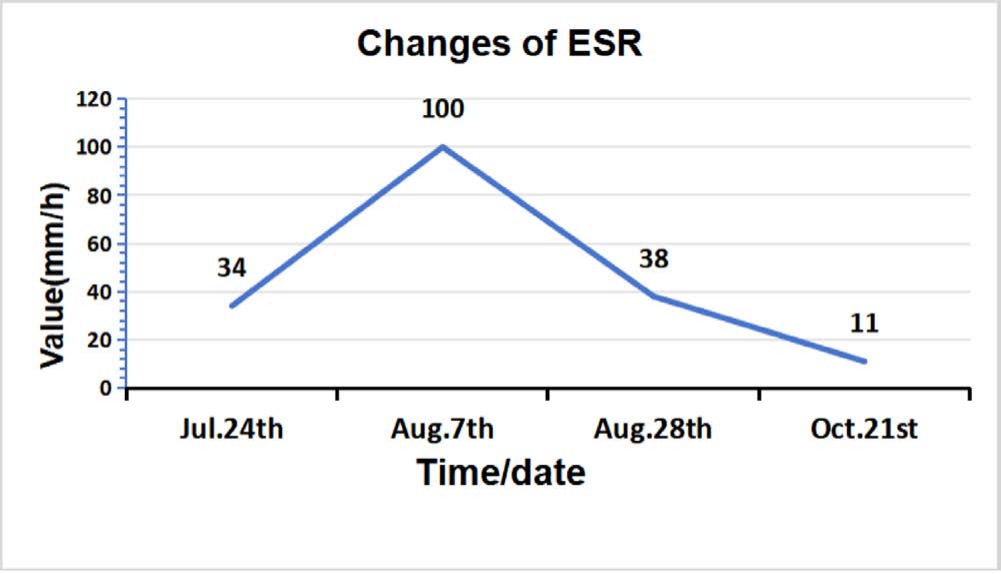
ESR returned to baseline. ESR = erythrocyte sedimentation rate.

**Figure 5.**
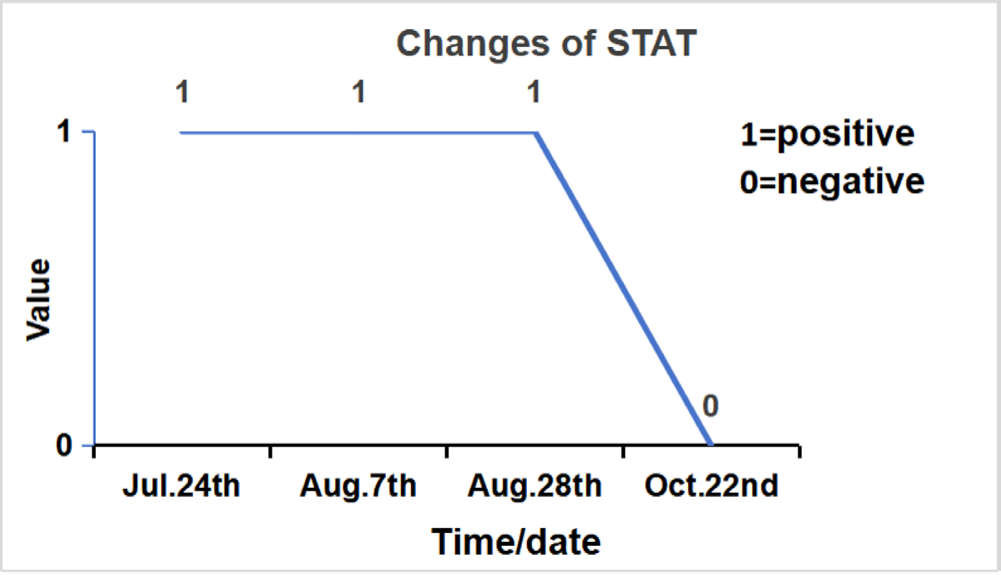
STAT converted to negative. STAT = standard tube agglutination test.

Seeking further management, the patient was admitted to our hospital on July 23, 2024. Throughout the course of her illness, she denied fever, sweating, fatigue, or other systemic symptoms. Physical examination revealed a longitudinal surgical scar over the anterior aspect of the left knee. A 1 × 1 cm sinus tract was observed inferolaterally to the incision, exuding necrotic material and clear yellow fluid. Mild swelling was present without localized erythema or increased skin temperature. Knee extension was full, flexion limited to 90°. The right knee showed no swelling, 20° valgus alignment, 20° extension lag, 105° flexion, and medial tenderness. The left hip showed no swelling. Patrick test was positive, indicating limited hip motion. Admission laboratory investigations: WBC 4.22 × 10⁹/L; IL-6 2.76 pg/mL; PCT 0.022 ng/mL; ESR 34.0 mm/h; CRP 4.43 mg/L. Repeated *Brucella* serological tests were positive: Rose Bengal agglutination test (RBT) positive, standard tube agglutination test (STAT) positive. Full-length standing anteroposterior radiographs of the lower limbs showed that the left knee prosthesis was stable without loosening or migration. The right knee demonstrated varus alignment and joint space narrowing. The left hip exhibited periarticular osteophytes and increased density of the articular surfaces (Fig. 2). Further history-taking revealed a 10-year history of contact with sheep. Positive *Brucella* serology, combined with repeated negative cultures for common bacteria at previous institutions, led to a diagnosis of *Brucella* infection of the left TKA prosthesis. Radiographic assessment confirmed stable components without signs of loosening. Given the patient’s preference for conservative management, antimicrobial therapy with doxycycline (200 mg once daily) and rifampicin (450 mg once daily) was administered for 12 weeks. Within 2 weeks of initiating therapy, wound drainage ceased, and the sinus tract showed near-complete closure. Significant improvement in knee pain and range of motion was noted (Fig. 3). At the 3-month mark, the sinus tract had fully healed. The knee was non-swollen, non-tender, and demonstrated normal range of motion. Serial ESR measurements progressively normalized (Fig. 4), and the STAT converted from positive to negative (Fig. 5). The patient has remained free of recurrence during 10 months of follow-up. The treatment timeline is summarized in (Table 1).

**Table 1:** Treatment course.

Date	Culture	STAT	Treatment
May 24, 2023	Negative	No	Underwent TKA. Received postoperative anti-inflammatory and analgesic therapy for 5 mo.
October 28, 2023	Negative	No	Underwent knee joint open incision and drainage. Commenced anti-tuberculosis therapy for 2 mo.
January 8, 2024	Negative	No	Underwent surgical debridement and drainage, polyethylene liner exchange, and continued anti-tuberculosis therapy for 7 mo.
July 23, 2024	Negative	Positive	Commenced oral doxycycline and rifampicin therapy for 3 mo.

STAT = standard tube agglutination test, TKA = total knee arthroplasty.

## 3. Discussion

*Brucella* infection causes a zoonotic infectious disease, endemic primarily in the northern provinces of China.^[2]^ The primary routes of *Brucella* transmission include ingestion of contaminated dairy or meat products, and direct contact with excretions from infected animals during abortion or parturition. Occupational exposure, particularly among butchers, laboratory personnel, hunters, and veterinarians, also constitutes a significant risk factor for infection.^[9]^ The patient in this case resides in northwestern China and had a prolonged history of contact with sheep flocks, placing her in a high-risk category for *Brucella* infection.

Brucellosis demonstrates persistent characteristics akin to tubercular infections. *Brucella* microbes can infiltrate host cellular environments (including phagocytic cells like macrophages), sustaining prolonged intracellular proliferation while obstructing phagocytic processes, eluding immunological detection, and impairing antimicrobial efficacy, ultimately resulting in sustained pathogenic colonization.^[10]^ Brucellosis frequently simulates diverse pathological states encompassing infectious and noninfectious categories such as rheumatic pathologies, inflammatory arthritides, enteric fevers, mycobacterial infections, parasitic diseases, degenerative joint disorders, and spinal abnormalities. The primary presenting features typically encompass febrile episodes, diaphoresis, asthenia, osteoarticular discomfort, muscular aches, lymphoreticular enlargement, and visceromegaly involving hepatic/splenic systems. In chronic disease progression, constitutional manifestations tend to be nonspecific, frequently characterized by generalized weakness, subjective unwellness, and musculoskeletal complaints. Focal manifestations of *Brucella*-associated prosthetic joint infections predominantly involve periarticular erythema, edema, local hyperthermia, nociception, and functional impairment. Clinical evaluation typically demonstrates elevated periarticular temperature gradients, palpation sensitivity, detectable synovial effusion through ballottement maneuvers, and compromised articular mobility.^[4,5]^

Bacterial culture is considered the “gold standard” for the laboratory diagnosis of brucellosis, exhibiting inter-study detection capability variances spanning 10% to 90%.^[11]^ A review by Faddoul L of 46 brucellosis patients from 3 Lebanese hospitals compared the sensitivity of different diagnostic modalities: RBT 94.7%, blood culture 65.6%, and SAT 95.1%.^[12]^ The RBT, IgG/IgM enzyme-linked immunosorbent assay, and polymerase chain reaction all demonstrate comparably excellent diagnostic performance. It is essential to emphasize that factors beyond diagnostic accuracy (such as accessibility, cost, and ease of operation) must be integrated into decision-making. The RBT demonstrates notable operational simplicity and cost efficiency, rendering it particularly advantageous in endemic areas of human brucellosis with constrained laboratory infrastructure. Enzyme-linked immunosorbent assay methodologies benefit from extensive commercial accessibility and high automation capabilities, thus enabling efficient screening of large patient cohorts. Conversely, polymerase chain reaction techniques continue to face standardization challenges, as evidenced by heterogeneous target selection and divergent protocols across studies, necessitating stringent procedural validation to achieve adequate detection thresholds.^[13]^ STAT specifically identifies immunoglobulin responses targeting the smooth cell wall lipopolysaccharide antigen demonstrated by *Brucella* pathogens, maintaining status as the principal serodiagnostic modality for brucellosis surveillance. Current diagnostic protocols mandate interpretation standardization where titers reaching or exceeding the 1:160 threshold in immunocompetent hosts, combined with corroborative clinical correlation, constitute validated diagnostic confirmation criteria for establishing active brucellosis infection.^[14]^ The sensitivity of the STAT for *Brucella* is significantly higher than that of bacterial culture, and it is also faster and more convenient. In the present case, the patient exhibited atypical systemic symptoms, with no fever, sweating, or fatigue. Physical examination findings of knee swelling, tenderness, and restricted range of motion were nonspecific. Laboratory investigations revealed normal WBC, PCT, IL-6, and CRP levels. Multiple cultures of joint fluid, wound discharge, and blood were negative. Only the ESR was mildly elevated. Imaging findings suggestive of effusion, synovitis, and soft tissue swelling were likewise nonspecific. Consequently, the patient was misdiagnosed with TB infection at other institutions. Therefore, we strongly recommend that routine *Brucella* serological screening should be performed for patients with PJI in brucellosis-endemic regions to exclude *Brucella* infection.

For the treatment of *Brucella* joint infections, dual antimicrobial therapy with doxycycline and rifampicin, or triple therapy with doxycycline, rifampicin, and streptomycin, is recommended for a minimum of 12 weeks.^[3,15]^ When PJI is identified following joint replacement surgery, in scenarios where imaging demonstrates stable implant fixation, conservative management involving antibiotic regimens alone may represent a feasible therapeutic approach.^[16]^ In cases where prosthetic instability (manifesting as loosening or malalignment) is radiologically confirmed, radical debridement with implant revision alongside culture-guided antimicrobial regimens becomes obligatory. Contemporary evidence supports comparable efficacy between single-procedure exchanges and interval-based staged protocols, with outcome optimization dependent on individualized time-window selection.^[16]^

This patient underwent TKA for left knee osteoarthritis in 2023. Full-length standing radiographs of the lower limbs demonstrated osteoarthritis in both the right knee and left hip (Fig. 2). Given the high prevalence of osteoarthritis among brucellosis patients,^[4,5]^ whether the underlying cause of her chronic peripheral polyarthritis was chronic osteoarticular brucellosis rather than primary osteoarthritis remains debatable.

Considering the prerequisites for knee arthroplasty, preoperative inflammatory markers such as CRP and ESR were presumably negative, indicating no overt active infection, which complicated preoperative exclusion of brucellosis. Upon presentation to our institution, the patient had experienced 13 months of persistent pain, sinus tract drainage, and functional impairment following TKA. Previous interventions, including arthrotomy with drainage, exchange of the tibial polyethylene insert, and antitubercular therapy at local hospitals, had failed. Multiple cultures of knee aspirates failed to identify a causative pathogen. However, her history of prolonged sheep contact and positive STAT confirmed the diagnosis of *Brucella* infection in the prosthetic knee joint. Given the absence of radiographic loosening and the patient’s strong preference for conservative management, she was treated with doxycycline (200 mg once daily) and rifampicin (450 mg once daily) for 12 weeks. As shown in Figures 3 to 5, at 2 weeks: the sinus tract was nearly closed with no drainage. Knee pain and restricted motion significantly improved. ESR remained elevated; STAT positive, at 4 weeks: the sinus tract healed completely. The knee was non-tender, non-swollen, and exhibited normal range of motion. ESR remained elevated; STAT positive, at 12 weeks: ESR normalized; STAT became negative, the patient has remained free of recurrence during 7 months of follow-up, demonstrating excellent therapeutic efficacy.

A sinus tract is a key clinical manifestation and diagnostic criterion for PJI; it is a pathological channel that forms between the skin and the joint, commonly observed in chronic PJI cases. Sinus tracts are associated with PJI caused by various microorganisms.^[17]^ The management of PJI following total knee arthroplasty encompasses debridement, antibiotics, and implant retention, as well as 1- or 2-stage revision arthroplasty. Debridement, antibiotics, and implant retention represents a preferable initial intervention for patients with stable, well-fixed components without evidence of sinus tract formation, who present with acute early postoperative or acute hematogenous infections. One-stage exchange or 2-stage exchange arthroplasty is the most common and beneficial treatment for PJI involving antibiotic-resistant organisms, sinus tracts, or compromised soft tissue coverage.^[18,19]^ Based on current clinical research and reports, *Brucella* PJI complicated by abscess/sinus tract formation in patients with stable implants has been managed with either debridement plus antimicrobial therapy or prosthesis exchange plus antimicrobial therapy. This case represents the 1st documented instance, to our knowledge, of a *Brucella*-infected TKA with an associated sinus tract and stable components successfully cured with antimicrobial therapy alone. Symptomatic relief was rapid and significant. This approach substantially reduced hospitalization time, medical costs, and surgical risks, and accelerated the return to normal activities for patients. Therefore, for patients definitively diagnosed with *Brucella* PJI following knee arthroplasty and radiographically stable components, conservative management with antimicrobial therapy alone should be considered a viable option, regardless of the presence of a sinus tract, potentially avoiding the need for prosthesis removal and spacer implantation.

*Limitations*: firstly, the findings are derived from a single case report, which inherently limits the generalizability of the conclusions. Secondly, the follow-up duration, although covering 10 months without recurrence, remains relatively short. Long-term surveillance is essential to confirm the durability of the treatment response. Future research should aim to include larger patient cohorts, longer follow-up periods, and standardized diagnostic protocols to validate the feasibility of antimicrobial-only therapy for *Brucella* PJI with sinus tracts and stable implants.

## 4. Conclusion

For patients in brucellosis-endemic regions who develop PJI, routine *Brucella* serological screening is recommended to exclude *Brucella* infection. For patients definitively diagnosed with *Brucella* PJI after TKA and with radiographically stable components, antimicrobial therapy alone may be considered as a viable conservative management option, regardless of the presence of a sinus tract, potentially avoiding the need for prosthesis removal and spacer implantation at the initial stage.

## Acknowledgments

We thank the patient for agreeing to submit his case.

## Author contributions

**Conceptualization:** Hongyin Fu.

**Investigation:** Song Sun, Xiuyuan Wang.

**Project administration:** Hu Chen, Rui Bai.

**Software:** Hu Chen, Honghao Fu, Libo Zhao.

**Writing – original draft:** Hongyin Fu, Song Sun.

**Writing – review & editing:** Hongyin Fu, Lianggong Zhao.
